# The analysis of SUDEP forensic autopsies leading to preventable events

**DOI:** 10.3389/fneur.2023.1231515

**Published:** 2023-06-29

**Authors:** Antonina Argo, Maria Puntarello, Ginevra Malta, Roberto Buscemi, Giovanni Scalzo, Valentina Triolo, Giuseppe Davide Albano, Stefania Zerbo

**Affiliations:** ^1^Department of Health Promotion, Mother and Child Care, Internal Medicine and Medical Specialties, Section of Legal Medicine, University of Palermo, Palermo, Italy; ^2^Policlinic Hospital, University of Palermo, Palermo, Italy

**Keywords:** SUDEP, epilepsy, forensics, autopsy, research, preventable events, artificial intelligence

## Abstract

**Introduction:**

The diagnosis of unexpected death by excluding non-natural causes, particularly in subjects with epilepsy, is a topic of interest and it is difficult to identify in the forensic field. Health professionals sometimes are faced with cases of sudden death, generally in young adults with a long history of epilepsy that require, for judicial purposes, an explanation in terms of cause and means to determine the death. SUDEP is an entity diagnosed by the exclusion of other causes that may have led to death, and then for forensic purposes, it requires particular attention and knowledge, and there is difficulty in identifying it. Our contribution aims to illustrate the scientific community pathological findings, medical history, and circumstantial evidence of four cases of sudden death in epileptic subjects.

**Method:**

We illustrated four cases of judicial autopsies from the Institute of Forensic Medicine of Palermo, Italy; the purpose was to exclude the criminal intervention in determining the death as non-natural. The study of victims’ medical history, the toxicological investigations, and the autopsy findings analyzed both from macroscopic and microscopic aspects have made it possible to highlight some findings that can be traced back to SUDEP despite the small sample of subjects studied.

**Results:**

These presented findings of four SUDEP cases could help forensic pathologists in recognizing this entity, by highlighting its characteristics, and allowing for a pathological classification, also in relation to the use of drugs for epilepsy treatment and circumstances of death.

**Discussion:**

To obtain a definite diagnosis of SUDEP, a complex investigation process is required in a multidisciplinary approach. Considering the literature review with criticism, it could allow health professionals to select the characteristics of epileptic patients at risk of sudden death. Processing human behaviors, molecular and histopathological findings of the autopsies, but also the physiological, and pathological human body system functions thanks to Artificial Intelligence, could be the key to explaining SUDEP mechanisms and the future results to prevent it.

## Introduction

**Figure 1:**
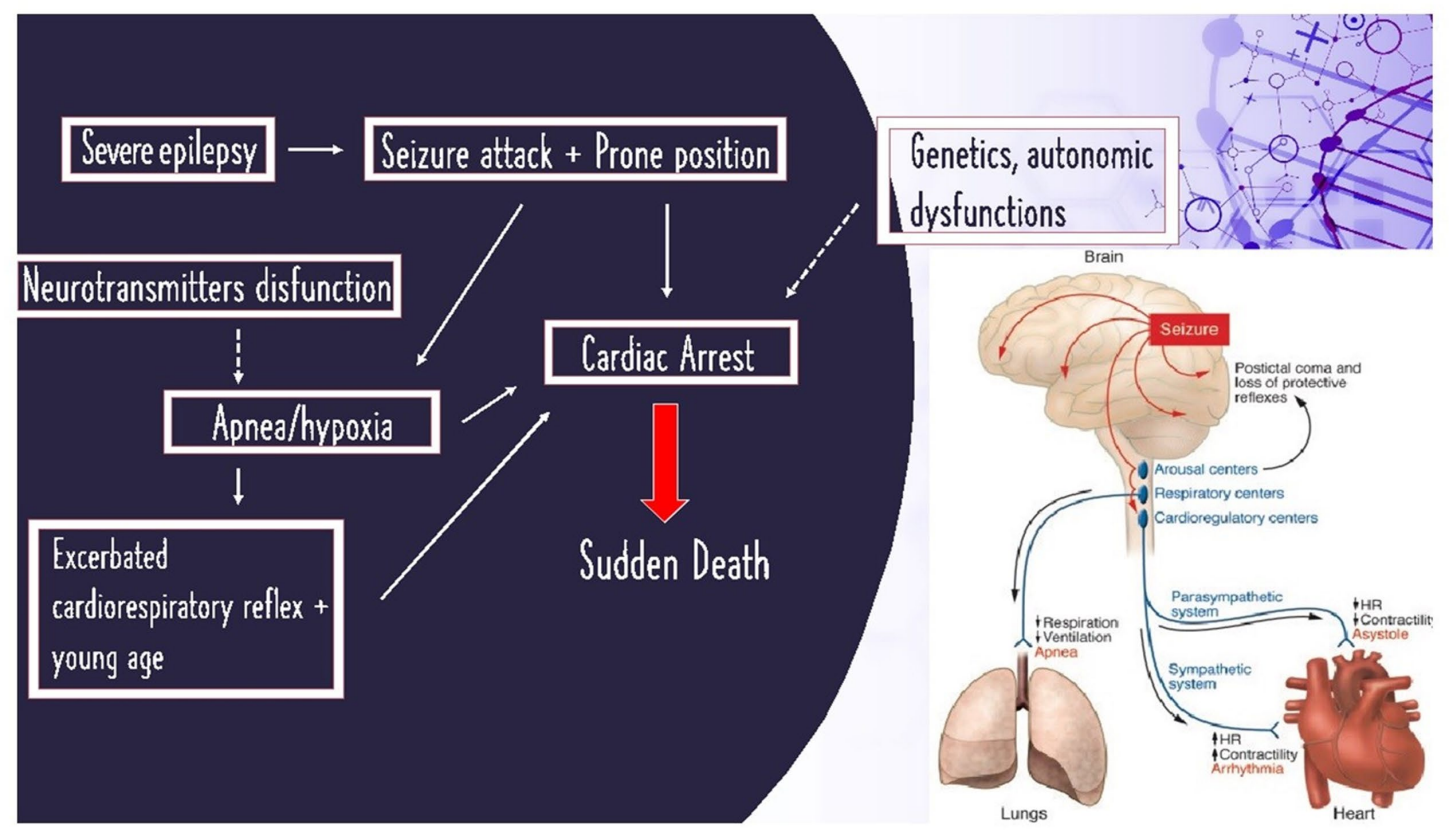
On the left: schematized histopathological mechanisms of SUDEP; on the right: image illustration of SUDEP from Friedman et al. (40).

Sudden cardiac death and sudden unexpected epilepsy death are the two major causes of sudden unexplained deaths (1). SUDEP is defined as “sudden, unexpected, witnessed or unwitnessed, non-traumatic, and non-drowning death in patients with epilepsy with or without evidence of a seizure and excluding documented status epilepticus, in which postmortem examination does not reveal a toxicologic or anatomic cause of death,” and it is classified into definite, probable, and possible (2, 3). In definite SUDEP, an autopsy has confirmed the absence of an anatomical or toxicological cause of death (4). In probable SUDEP, an autopsy has not been done but the circumstances of death are strongly suggestive of SUDEP, while possible SUDEP describes a situation in which SUDEP cannot be excluded and should be considered among the explanations of death (5–7). Epilepsy is a common neurological disorder with population rates ranging from four to 10 per 1,000 people. It is characterized by seizures. More than 50 million people worldwide have epilepsy. Epileptic people have a 2–3 times higher risk of premature death than the general population, and the risk of sudden and unexpected death is approximately 24 timers higher (8, 9). Sudden and unexpected death in epilepsy (SUDEP) represents the main cause of premature deaths in young adults (between 20 and 40 years of age) suffering from epilepsy (10–12). It is more common in patients with poorly controlled generalized seizures (13). To date, the pathological mechanisms of SUDEP are unknown and unclear. Several studies indicate that tonic-clonic seizures can lead to transient respiratory arrest and apnea. The extent of oxygen desaturation is related to the convulsion’s duration time, and it is associated with an increase in end-tidal carbon dioxide levels. Authors have shown that seizure activity can cause hypoventilation and, therefore, hypoxemia and hypercapnia (13). Suspected SUDEP’s mechanisms also include changes in cardiovascular stability and baroreflex sensitivity during the interictal state. Seizure activity can also be associated with acute pulmonary edema from increased pulmonary vascular pressure and central apnea that result in fatal anoxia (14). This evidence suggests that a combination of acute cardiovascular and pulmonary events related to epileptic discharges may cause death (9, 15, 16). An elevated seizure frequency is a risk factor for SUDEP. An interesting study by F. Scorza et al. aims to share with the scientific community the possible correlation between aberrant neurogenesis of epileptic patients and seizure frequency. Based on these results, the aberrant neurogenesis could negatively influence the cardiovascular system of the patient with epilepsy, leading to cardiac abnormalities and, therefore, SUDEP (17). Several studies have identified that some drugs, antiepileptics, for example, could determine an arrhythmic death (18–20) or induce acquired long-QT syndrome (21). Although the histopathological findings in death related to SUDEP are unclear, Theodora A. Manolis tried to explain the possible mechanism of SUDEP, highlighting respiratory and cardiovascular dysfunction as potential mechanisms of sudden death in epileptic patients as well as the disruption of the central autonomic control in SUDEP (22). Recently, several studies using imaging with magnetic resonance and measurements of heart rate variability suggested that a dysfunction of the brainstem could increase SUDEP risk (23). Several postmortem studies reported that disorganization of the hippocampus and amygdala that appears with altered gray matter volumes on MRI has a role in the control of the autonomic nervous system (24) and may increase the risk of SUDEP (25). In addition, cardiovascular dysfunction plays an important role in the determination of SUDEP: in epileptic patients, we could highlight arrhythmias, bradyarrhythmias, or tachyarrhythmias related to epileptic drugs. The correlation between drugs (lamotrigine and carbamazepine) is discussed (26). This is an important topic to highlight because some epileptic patients take two or more different antiepileptic drugs to control seizures, and a lot of study demonstrates that polytherapy is a risk factor for SUDEP (23, 27). Variable compliance with antiepileptic drugs could be a potentially preventable cause of sudden unexpected death in epilepsy. To confirm the correlation between a low level of drugs and SUDEP during an autopsy, it is important to make hair analysis and blood/urine laboratory exams (28). Recent studies by the scientific community described a possible pathological mechanism of SUDEP related to some neurotransmitters, such as serotonin and adenosine. Adenosine is an inhibitory modulator of neuronal excitability; while adenosine increases neuronal activity, serotonin can modulate neuronal excitability, stimulating respiratory centers in response to hypercapnia. Indeed, MRI-based measurement showed that the brain volume of the medullary raphe (29), where serotonin is produced, was lost in patients that died of SUDEP. During and after seizures, the rise of adenosine is correlated to respiratory failure (30). Therefore, these neurotransmitters provide possible treatment targets for SUDEP (30). Lastly, genetic alterations were found in patients with SUDEP diagnosis (31). Germline loss-of-function mutations in DEPDC5 cause focal epilepsies and increase SUDEP risk (32). Emerging genetic research suggests a correlation between mutations in ion channel genes and familial LQTS and SUDEP (33) and variants of KCNQ1, KCNH2, and SCN5A genes (34–37). Patients with sodium channel mutations are predisposed to progress from mild cerebral edema to severe cerebral edema which may represent an additional contributing factor in the events leading to the sudden death of patients with epilepsy (38, 39). The complexity of the histopathological mechanisms of SUDEP is simply schematized in Figure 1.

## Materials and methods

Our contribution aims to share with the scientific community pathological findings, medical history, and circumstantial evidence of four cases of sudden death in epileptic subjects. These are four cases at first judicial autopsies from the Palermo’s Institute of Forensic Medicine in which the purpose was to exclude criminal intervention in determining the death as non-natural. The study of the patients medical history, the toxicological investigations, and the autopsy findings are analyzed from macroscopic, histopathological, and toxicologic findings. All forensic blood samples were screened for alcohol, drugs, and medicinal drugs. Ethanol was screened and quantified by headspace gas chromatography methods combinate with flame ionization detection. Illegal or medical drugs were screened by an immunological method with confirmation and quantification using gas-chromatography mass spectrometry.

### Case 1

A male subject, aged 33, who was a prisoner. He was found in the early-morning hours unconscious and pulseless while asleep in bed. He featured a clenched jaw, the tongue clamped between his teeth, and he was drooling. In his medical history, there was the presence of psychiatric disorders and drug addiction but not epilepsy. He was taking 1,500 mg daily. The external examination showed a mantle-like congestion of the cranio-cephalic district, the tongue stapled between the teeth with a dental impression to the left of the tip, abrasions at the upper and lower gingival fornices, and subungual cyanosis. His brain’s weight was 1,114 g. Macroscopically, an edematous encephalon with flattened circumvolutions and hemorrhagic punctuation was observed, which was also confirmed on histopathological examination. In the cerebellum, there were histological signs of hemorrhagic areas and neuronal necrosis, while in the bulb and medulla, evidence of neuronal edema alternating with areas of ischemic distress was detected. The lungs weighed 1.690 g; they were edematous with macroscopic and microscopic signs of acute pulmonary edema; the heart weighed 310 g and had diffused hemorrhagic petechiae at the pericardial and epicardial surfaces at the tip and posterior surface. Toxicological examinations showed the presence of valproic acid within the cut-off. The autopsy did not reveal an anatomic or toxicologic cause of death; there were no findings of criminal interventions, and signs of typical indirect seizures were found, which allowed for the diagnosis of SUDEP.

### Case 2

A male subject, aged 41, with a positive medical history of epilepsy with tonic–clonic seizures and alcohol and drug addiction. He was found in the early-morning hours unconscious and pulseless while asleep in bed. He was taking 100 mg of phenobarbital daily. Upon external inspection, there was cranio-cephalic congestion; upon opening his mouth, there was the presence of a protruded and stapled tongue between the teeth and a foamy material inside it. Approximately 2 days earlier, he had a seizure with respiratory failure. His brain’s weight was 1,115 g, his lungs weighed 1,550 g, and his heart weighed 350 g. Macroscopic examination showed brain edema, pulmonary congestion, and left ventricular hypertrophy. The left ventricular free wall measured 1.5 cm, while the right ventricular free wall was 0.5 cm. The septum measured 1.2 cm. Microscopically, significant cerebral and pulmonary edema was confirmed. Toxicological analysis showed positivity to barbituric and benzodiazepines within the cut-off. Microscopic and histopathological findings excluded anatomic or toxicological causes of death.

### Case 3

A female subject, aged 67, with a positive medical history of epilepsy and psychiatric disorders. She was being treated with valproic acid of 1,500 mg daily and escitalopram. She was found in the afternoon dying. On reaching the emergency room, her death was noted. On external inspection, there were no indirect signs of seizures and evidence of cervical-cephalic congestion and subungual cyanosis. At autopsy, the leptomeninges appeared opaque; the encephalon was diffusely edematous, and its weight was 1,010 g. The heart weighed 350 g; it was flaccid to the touch, with evidence of hemorrhagic spiking at the free wall of the right atrium. Its transverse diameter was 10.5 cm, and its longitudinal diameter was 14 cm. The left ventricular free wall was 1.3 cm, the right ventricular free wall was 0.3 cm, and the septum measured 1.3 cm. The lungs were mildly edematous. Microscopically, massive cerebral edema and neuronal reduction of the bulb and medulla were confirmed. The lungs weighed 1,185 g and showed signs of blood stasis with areas of fibrosis and focal edema. All toxicological analyses were negative. Anatomic or toxicological cause of death was not found.

### Case 4

A 16-year-old female subject with intellectual disability and epilepsy was a resident of a nursing facility. The patient was treated with valproic acid of 1,000 mg (morning and evening), Perampanel 10 mg (evening), and diazepam 10 drops (morning and evening). Early in the morning, the girl was found dead, and an autopsy was ordered. On external examination, cervical and encephalic congestion was noted. On examination of the organs, the brain appeared edematous, with flattened furrows and congestion of the leptomeningeal vessels; the heart, weighing 375 g, showed the following thicknesses: the left ventricle was 1.4 cm, the interventricular septum was 1.1 cm, and the right ventricle was 0.8 cm; the lungs weighed 1.670 g and diffuse edema was evident on cutting surface. First-level toxicological screening tests were performed on blood matrix which showed positivity for benzodiazepines, which was compatible with the drugs in use. Intellectual disability worsened the girl’s reflex response to seizures. Her clinical history was complicated by drug-resistance epilepsy. It required polytherapy drugs.

## Discussion

SUDEP is the most common cause of death in epileptic patients. In forensic field making, a diagnosis of SUDEP is difficult (14, 41). As the diagnosis of SUDEP is also made by the exclusion of other causes of death, forensic pathologists must collect informations about the death scene, the circumstance of the death, and the victim’s medical history. Recent clinical findings and symptoms before the death must be sought although prodromal symptoms are often non-specific (42). The type of drugs eventually taken by the victim could link with sudden death but the external examination by the forensic pathologist could highlight signs and findings that indirectly link to epilepsy, such as abrasions and/or ecchymosis in areas of accidental fall trauma (protruding areas of the face, extensor surfaces of limb joints, conjunctival petechiae, lacerations, or hemorrhagic infiltration of the tongue) (21). Generally, sudden death, especially in young people always requires a systematic forensic autopsy including toxicological analyses. Sudden unexpected death in epilepsy is an interesting topic for forensics. Indeed, a lot of studies and scientific literature come from retrospective postmortem studies carried out by schools of Legal Medicine (12). Luo Zhuo and his colleagues highlighted autopsy cases of SUDEP in the Office of Chief Medical Examiner, in the State of Maryland (41). From 2007 to 2009, they analyzed 104 cases of sudden unexpected deaths directly or indirectly caused by epilepsy or seizures. Their findings are similar to our cases: subjects’ prevalence in age was between 21–50 years (41), death was especially during early-morning hours, on external examination there were indirect findings of seizure, and on microscopical findings, there were cerebral and pulmonary edema and neuronal necrosis. Our case history although small deviates from the literature because we had an equal incidence between male and female SUDEP victims. An interesting item to analyze to properly diagnose, even in forensic and histopathological fields, is the victims’ risk factor for SUDEP (43, 44). Risk factors are today’s study objects and not of unequivocal interpretation: the scientific community generally agrees on young subjects and male subjects, with epilepsy diagnosis at an early age and long illness duration, with tonic–clonic and nocturnal seizures and with antiepileptic polytherapy or epilepsy non-medication treatment. Alcohol and drug consumption increases SUDEP risk (6, 43, 44). For forensics, an interesting question is the relationship between seizures and sudden cardiac arrest: could the seizure be a trigger for sudden cardiac arrest? Often, forensic pathologists highlight indirect findings of seizures on the victims, but such evidence, although suggestive, is not absolutely linked to death during a seizure (45). This aspect is, indeed, non-clear in the scientific literature. E.C. Stecker and colleagues analyzed a population with epilepsy and sudden cardiac arrest; in 66% of epileptic patients, there was no relationship between seizures and sudden cardiac arrest (27). On the other hand, studies affirmed the claim otherwise (46, 47). By analyzing our findings, both macroscopical and histopathological signs and circumstantial details are consistent with the literature study (48–54): in fact, all four cases showed cerebral and pulmonary edema, and seizure was not directly seen by witnesses; in two of four cases, there were indirect signs of seizures, and an altered toxicological range of anti-epileptic drugs was not found. Three patients took monotherapy for epilepsy, while only one patient took polytherapy, and two victims were drug/alcohol addicted. There were no cardiac fibrosis findings in the histopathological study (55). The schematic representation of our findings is included below (Table 1).

**Table 1:** Macroscopic and histopathological findings in our SUDEP death diagnosis.

	Gender	Age	Epilepsy diagnosed	Polytherapy for epilepsy	Type of molecular therapy	Indirect signs of seizures	Death in bed	Cerebral/pulmonary edema	Neuronal necrosis
Case 1	Male	32	No	No	Valproic Acid	Yes	Yes	Yes	Yes
Case 2	Male	41	Yes	No	Barbituric	Yes	Yes	Yes	No
Case 3	Female	67	Yes	No	Valproic Acid	No	Unknown	Yes	Yes
Case 4	Female	16	Yes	Yes	Valproic Acid perampanel diazepam	No	Yes	Yes	n.d.

**Figure 2:**
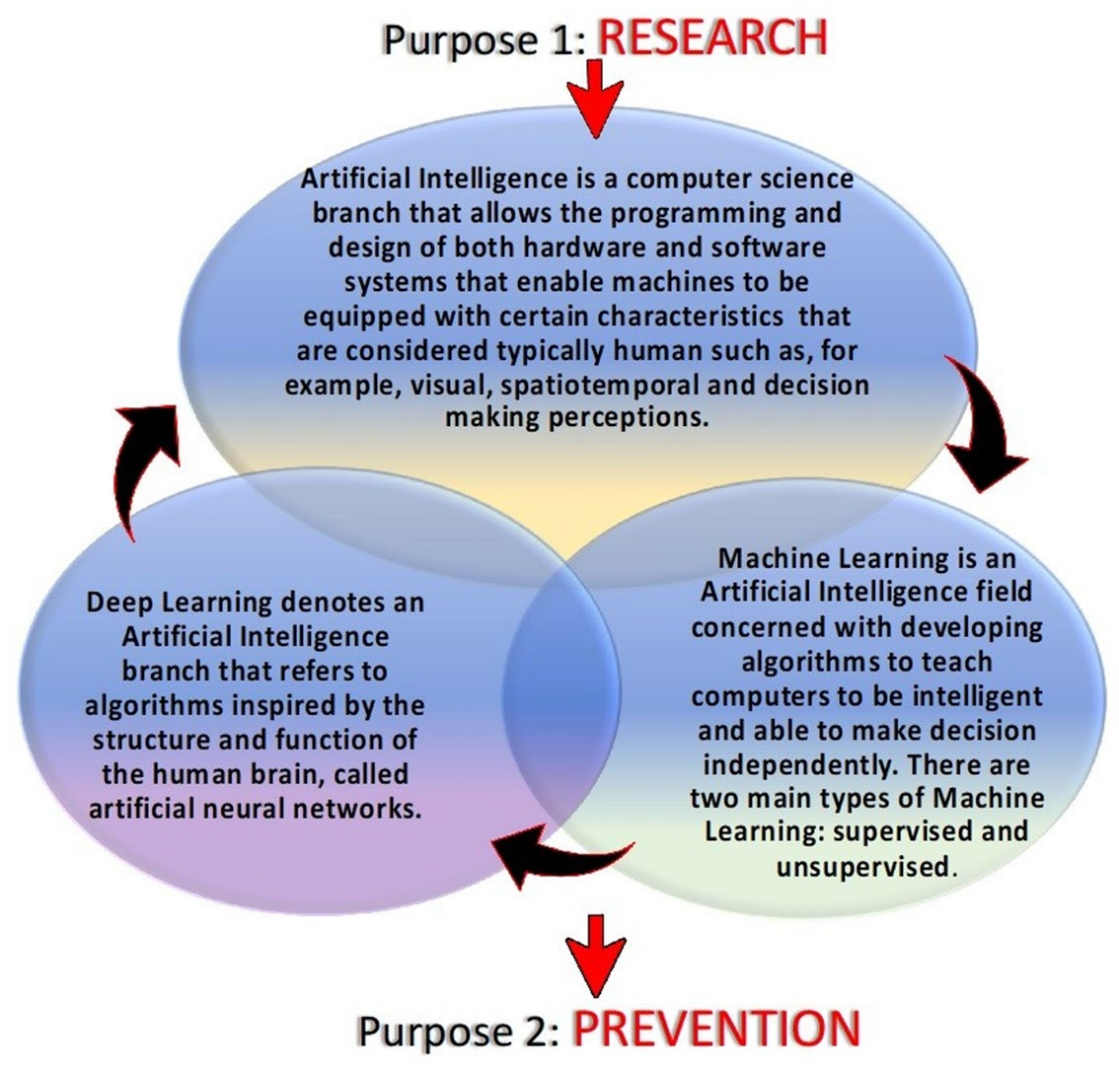
The use of artificial intelligence and its subtypes.

A forensic autopsy could highlight SUDEP characteristics and facilitate SUDEP diagnosis by analyzing many aspects simultaneously: circumstantial details, death scene, external inspection, macroscopical and microscopical findings, and toxicological investigations. These elements make it possible to have a definite diagnosis of SUDEP. This process of diagnosis is complex and requires a multidisciplinary approach. When more than one pathological finding is found in a case of suspected SUDEP, making a differential diagnosis becomes extremely difficult. We aim to detect alterations that, with the support of further studies and numbers of SUDEP, can highlight specific features that can more easily be attributed to SUDEP. The autopsies’ pathological findings alterations found that, if individually taken into consideration for critical appreciation without the contribution of circumstantial data, indirect signs of a seizure and medical history are not specific elements, which is why identifying a diagnosis of SUDEP is not easy. For this reason, in the future perspective, it would be desirable to identify markers that if dosed make the autopsy evidence more specific. In this regard, an important role could be played by heat shock protein 70 (HSP70), which is a molecular chaperone involved in the inflammatory response that is upregulated after the epileptic state. HSP70 has been described as an endogenous intracellular ligand of Toll-like receptor 4. It is released from damaged tissues and activates immune cells after an epileptic seizure. The timing and mode by which HSP70 is released are unclear to date. There are not many human studies in the literature; something has been found in an animal study, indicating the overexpression of HSP70 immediately after seizure (56). Studying the immunohistochemical expression of HSP70 in the hippocampus, the parahippocampal cortex, parietal cortex, amygdala, and thalamus (areas most affected by neuronal damage during a seizure) could not only allow us to understand whether the timing of seizure is closely related to SUDEP, but in the forensic setting, positivity to HSP70 could bring important implications in the safety diagnosis of SUDEP by making macroscopic and histopathological evidence more specific. An additional benefit could be to make HSP70 a therapeutic target to limit the neuronal loss and inflammatory reaction control (4, 57–60). Automatically, this would reduce the frequency of seizures and could be a protective factor for SUDEP in patients most at risk. In the future perspective, with the advancement of artificial intelligence in the medical field, it might be useful to create algorithms that based on the clinical characteristics of patients can identify those most at risk of sudden cardiac death so as to attempt experimental pharmaceutical approaches (target HSP70) and apply closer monitoring, especially during the night, a time when, statistically, SUDEP occurs most frequently based on the present state of the heart (57). Recent studies have focused attention on wearable multimodal bracelets, among them Embrace and E4, that are based on the detection of electrodermal activity, motion sensors, plethysmography, and temperature to detect crisis and through signaling mechanisms alert is sent to the rescuers (61). The wristbands use machine learning mechanisms facilitated by the user’s ability to report false alarms *via* the app (62, 63). The bracelets represent an evolution of audio-video monitoring during the night, an ambulatory method used for the study of nocturnal seizures (64). Other methods that could help healthcare providers identify seizures in the future could be evolutions of video-audio monitoring involving the use of previously programmed algorithms along with deep learning mechanisms (65, 66). The foundation of artificial intelligence is used to describe “machines” able to demonstrate cognitive functions that humans associate with other human minds such as learning and problem-solving. Machine learning is based on the compilation of a complex algorithm and software that mimics the human mind to decipher critical problems that include visual perception, decision-making, and speech recognition. Deep learning is similarly described as a class of artificial neural networks that learn in a supervised and unsupervised manner. To analyze real-world data, deep learning decomposes information into various abstraction levels. Each decomposition level corresponds to a neural network. Artificial intelligence can learn human behaviors in different areas. In medicine and particularly in the case of SUDEP, artificial intelligence and deep learning through supervised or unsupervised learning modes could allow us to act on two fronts. The first involves a study of the human activity of the epileptic subject to research the mechanisms underlying sudden cardiac death. For example, we could consider using deep learning to study the characteristics and variations of respiratory, cardiac, and nerve activity by decomposing each apparatus into various abstract sublayers. In this way, by monitoring all patients defined as “at risk,” we could highlight the pathophysiological mechanisms of SUDEP that today are unclear. The interaction between the various systems of the human body in the determination of sudden cardiac death in epileptic patients could be investigated by encouraging machine learning (67, 68). The second use of deep learning is closely related to the first: through machine learning resulting from research, extrapolated data can be applied for prevention purposes. The interaction of algorithms based on the study of respiratory activity (e.g., the number of breath acts, the depth of each breath, and the blood acid–base balance) but also the study of the various sub-levels of cardiac and nerve activity before, during, and after a seizure could lead to highlighting critical passages, a prelude to SUDEP, so as to promptly alert the rescuers and increase the likelihood of saving a patient as well as identifying which of them is actually most at risk, progressively limiting false alarms through the use of machine learning (Figure 2).

Processing human behaviors, molecular and histopathological findings of the autopsies, but also the physiological and pathological human body system functions thanks to Artificial Intelligence could be the key to explaining SUDEP mechanisms and the future results to prevent it.

## Conclusion

Attention to SUDEP pathophysiology, the study of physiological changes detected in SUDEP victims, cardiac study with ECG monitorization, post-mortem instrumental investigations such as CT or MRI, the study of the genetic predisposition for SUDEP, and the study of interactions between antiepileptic drugs and SUDEP could lead to important implications in the knowledge of the illness but especially in preventing sudden cardiac death in epileptic patients. To assess the state of knowledge about SUDEP, the American Epilepsy Society and the Epilepsy Foundation convened a task force that had five goals: develop a position statement describing if, when, what, and how SUDEP should be discussed with patients, their families, and caregivers; design methods by which the medical and lay communities become aware of the risk of SUDEP; recommend research directions in SUDEP; explore steps that organizations can take to perform large-scale, prospective studies of SUDEP to identify risk factors; identify possible preventive strategies for SUDEP (69). In the forensic field, often the purpose is excluding criminal intervention or non-natural death causes. SUDEP diagnosis is important, but with our small contribution, we aim to identify indirect and direct findings of SUDEP during autopsies that could facilitate SUDEP identification, knowledge, and prevention (70, 71). We aim to launch a hypothesis involving the collaboration of artificial intelligence and, in particular, deep learning and the study of HSP70 expression that could not only elucidate the pathophysiological mechanisms of SUDEP and its correction by seizures but also facilitate the autopsy diagnosis of SUDEP and intervene in the prevention of sudden unexpected death in epileptic patients.

## Data availability statement

The datasets presented in this article are not readily available because of restriction on judicial data and privacy restrictions. Requests to access the datasets should be directed to the corresponding authors.

## Ethics statement

The studies involving human participants were reviewed and approved by the Ethics Committee of the Policlinic Hospital "P. Giaccone". Written informed consent to participate in this study was provided by the patient/participants' legal guardian/next of kin.

## Author contributions

AA, MP, and GM designed the framework of the review and drafted the manuscript. SZ, GA, RB, GS, VT, and AA provided supervision and contributed to manuscript writing and editing. All authors have read and approved the latest version of the manuscript.

## Funding

This work was supported by University of Palermo, Eurostart 2021-22, cod. n. PRJ-1030. Title: “Tissue markers predictive of damage from substances of abuse and their correlation to preventable adverse cardiovascular events”.

## Conflict of interest

The authors declare that the research was conducted in the absence of any commercial or financial relationships that could be construed as a potential conflict of interest.

## Publisher’s note

All claims expressed in this article are solely those of the authors and do not necessarily represent those of their affiliated organizations, or those of the publisher, the editors and the reviewers. Any product that may be evaluated in this article, or claim that may be made by its manufacturer, is not guaranteed or endorsed by the publisher.
